# Improving Decision-to-Incision Interval (DDI) of Emergency Cesarean Sections Through Mobile-Based Obstetric Emergency System (MORES) and Midwife-Led Triage in Bong County, Liberia: A Quasi-Experimental Study

**DOI:** 10.3390/ijerph22101596

**Published:** 2025-10-21

**Authors:** HaEun Lee, Sunghae Kim, Joseph Sieka, Wahdae-Mai Harmon-Gray, Philip T. Veliz, Jody R. Lori

**Affiliations:** 1 School of Nursing, University of Michigan, Ann Arbor, MI 48109, USA; 2 College of Health Sciences, University of Liberia, Capitol Hill P.O. Box 10-9020, Monrovia 1000, Liberia

**Keywords:** emergency cesarean section, obstetric emergency, decision to incision, referral, midwife-led triage program, sub-Saharan Africa, mhealth

## Abstract

**Background**: Delays in emergency cesarean section (CS) remain a major contributor to maternal and neonatal morbidity in low-resource settings. This study evaluated the combined effect of a mobile-based obstetric emergency system (MORES) and a midwife-led triage program on the decision-to-incision interval (DDI) and related outcomes in Liberia. **Methods**: A quasi-experimental study with an interrupted time series design was conducted in Bong County across two district hospitals receiving referrals from 20 rural health facilities. Seventy-two women referred for emergency CS were observed at baseline, midline, and endline. MORES used WhatsApp-based communication to improve referral coordination, while the triage program trained midwives to rapidly assess cases using a color-coded system. Data were analyzed using descriptive statistics, Wilcoxon rank-sum, chi-squared tests, and logistic regression. **Results**: By endline, the median DDI decreased by 117.5 min compared to baseline (95% CI: −205.1 to −29.9). Women were significantly more likely to receive a CS within 75 min (AOR: 11.7; 95% CI: 1.32 to 104.5). No maternal deaths occurred. Neonatal mortality was observed but not significantly associated with DDI. **Conclusions**: MORES and midwife-led triage substantially improved the timeliness of emergency CS in a resource-constrained setting. These low-cost, feasible strategies warrant further evaluation for sustainability and impact on neonatal outcomes.

## 1. Introduction

Globally, the use of cesarean sections (CSs) has steadily increased as a critical tool in modern obstetrics, allowing for the safe delivery of both mother and child during complicated births [1,2]. Timely planning and early recognition to perform emergency CS is essential to prevent death and severe complications from obstetric conditions such as obstructed labor, uterine rupture, and fetal distress [3]. The World Health Organization (WHO) recommends that optimal CS rates be around 10–15% to reduce maternal and neonatal mortality, with rates above this threshold offering no additional benefit [4]. Although the global average CS rate has increased by 19% from 1990 to 2018 [5], sub-Saharan Africa (SSA) falls behind, with average emergency CS rates around 4.6% [6]. In Liberia, the national CS rate remains low, estimated at 5.3% in 2020 [7], highlighting major disparities in access to life-saving surgical obstetric care, particularly in rural and underserved regions.

These deficiencies are especially pronounced in settings where barriers such as poverty, low maternal education, lack of antenatal care, and limited health insurance restrict access to emergency obstetric services [8]. In 2023, approximately 70% of global maternal deaths occurred in SSA, and Liberia ranks among the highest with a maternal mortality ratio of 742 per 100,000 live births [7]. Inadequate access to timely CS contributes substantially to this burden. The decision-to-incision interval (DDI)—the time between the decision to perform a CS and fetal delivery—is a key metric in emergency obstetric care [9].

UK National Institute for Health and Care Excellence (NICE) recommend a DDI of ≤30 min for category 1 cases, which are immediate threat to the mothers or fetus’s life, including potential uterine rupture, placental abruptions, cord prolapses [10,11]. DDI of ≤75 min is recommended for category 2 cases that are compromising maternal and fetal health but not an immediate threat to life. Category 3 cases are when there is no compromise to mother or fetus but early birth is required and category 4 that suits the mother or medical professionals [10]. However, studies across SSA consistently report median DDIs of one to two hours or more [11,12]. A recent meta-analysis found that only 10.2% of emergency CS in Africa meet the 30 min DDI standard [13]. In Nigeria, Abdulbaki et al. (2024) found a median DDI of 200 min, resulting in elevated odds ratio [OR] for perinatal death (OR = 6.9), neonatal intensive care admission (OR = 9.8), and maternal complications such as sepsis and cardiac arrest [11].

In Liberia, health system constraints—including shortages of skilled staff, limited emergency readiness, and referral delays—contribute to prolonged DDIs and preventable maternal and neonatal harm [14,15,16]. The Service Availability and Readiness Assessment (SARA) conducted in 2018 showed that only 6% of Liberian facilities met full emergency obstetric and newborn care (EmONC) readiness criteria, and only 42% were equipped to make emergency referrals [15]. This systemic fragmentation hinders timely responses to obstetric emergencies and underscores the need for scalable, context-appropriate interventions.

To address these challenges, low- and middle-income countries (LMICs) have begun exploring low-cost, scalable innovations that improve triage and interfacility coordination. For example, mobile health interventions such as WhatsApp-based referrals have shown potential in improving communication and expediting emergency care [17,18] while adoptable interventions like midwife-led triage models and checklist-driven decision-making tools have improved identification and prioritization of high-risk cases [19,20,21]. Despite these promising developments, most research on referral interventions remains concentrated in a small number of countries, and evidence from real-world implementations remains limited—particularly in Liberia, where health system research and reform are urgently needed [20,22].

Emerging frameworks like the “Networks of Care” model emphasize the need for coordinated and people-centered systems to improve maternal health outcomes through better alignment of service delivery, infrastructure, and accountability [23,24]. In this context, strengthening referral pathways and triage practices can be especially impactful in bridging the gap between health centers and higher-level hospitals.

To contribute to this growing evidence base within LIMICs including Liberia, our study implemented and evaluated the impact of two complementary interventions: a. the Mobile-based Obstetric Emergency Referral System (MORES), a WhatsApp-based digital communication platform linking rural health facilities (RHFs) to district hospitals; and b. a midwife-led triage program implemented at district hospitals to improve rapid assessment and prioritization of obstetric emergencies. This paper aimed to assess the combined effect of these interventions on DDIs—across three categories (median, ≤30 min, and ≤75 min)—and associated neonatal outcomes in a Bong County, Liberia.

## 2. Material and Methods

### 2.1. Setting

This study was conducted in Bong County, Liberia, the third most populous county in Liberia with a population of approximately 467,502 residents (Liberia Census, 2022). In 2023, Liberia had a maternal mortality rate of 742 for every 100,000 live births, the fifth highest globally of that year [7]. Since 2013, coverage of prenatal care from skilled providers in Liberia has consistently exceeded 95%. Nurses and midwives are the primary providers, delivering care to more than three-quarters of pregnant women (78%), while doctors account for 19% of prenatal care visits [7].

In 2019, Bong County reported a total of 15,231 facility-based deliveries attended by skilled health personnel [7]. Of these, approximately 81% occurred at RHFs offering Basic Emergency Obstetric and Newborn Care (BEmONC), typically overseen by nurses or midwives [7]. These RHFs are typically staffed by nurses, midwives, and physician assistants. The treatments that BEmONC facilities offer include 7 functions: (a) administering parenteral antibiotics, (b) administering uterotonic drugs, (c) administering parenteral anticonvulsants, (d) manually removing the placenta, (e) removing retained products, (f) performing assisted vaginal delivery, and (g) performing basic neonatal resuscitation [25]. Because most RHFs are not equipped to perform surgical interventions, they rely on timely referrals to nearby district hospitals for cases of obstetric complications [26]. The remaining 19% of deliveries occurred at referral hospitals equipped with Comprehensive Emergency Obstetric and Newborn Care (CEmONC) services [7]. CEmONC includes all the 7 signal functions in BEmONC in aDDItion to performing surgery, such as CS, and performing blood transfusions (WHO, 2009). Approximately 84% of deliveries in Bong County take place in health facilities, and the county reports the highest rate of CS in Liberia, accounting for 8% of all births [7].

### 2.2. Interventions

The MORES intervention was implemented at two district hospitals—Phebe Hospital and CB Dunbar Hospital—and 20 RHFs that refer patients to these hospitals. Nurses and midwives from both the hospitals and RHFs participated in the training. The midwife-led obstetric triage intervention was implemented at the same two district hospitals, first training five midwives as “champion trainers” who in turn trained an additional 62 midwives at the two district hospitals.

The MORES training introduced healthcare providers to the intervention’s key components, including standardized WhatsApp messaging templates to facilitate referrals and handovers between RHFs and district hospitals. Each RHF was linked to its respective district hospital through dedicated WhatsApp groups to support real-time, bi-directional communication [27]. District hospitals were provided with smartphones exclusively for MORES use in their emergency departments, while RHF staff used their personal phones. Cellular scratch cards were distributed throughout the study to ensure consistent mobile data access [18]. Additional details on the intervention’s implementation, including pre/post odds of CS and maternal and newborn outcomes, are described elsewhere [27].

In parallel with MORES, a midwife-led obstetric triage program was implemented at the same two district hospitals. Originally developed by the Ghana Health Service and Kybele, Inc., a U.S.-based non-governmental organization [28], this program follows a train-the-trainer model. Hospital leadership nominated five midwives who completed an intensive two-day training to become triage champions. These champions subsequently trained 62 additional providers across the two hospitals [29]. The training covered rapid obstetric evaluation, emergency management, and a color-coded classification system (low, medium, high) to assess patients’ clinical conditions. The program aimed to strengthen district-level capacity to triage and manage referred obstetric patients upon arrival. Additional details on training content and changes in midwives’ knowledge scores are reported in Sieka et al. (2024). Both interventions were launched in March 2022 [29]. Additional descriptions of both the MORES and the midwife-led obstetric triage interventions are available elsewhere [18,27,29].

### 2.3. Data Collection

Structured observational data, using a systematic and predefined data collection protocol and tools, were collected by two experienced nurse-midwives trained research assistants (RAs) stationed in the labor wards of the two district hospitals. Because these were observational data, we did not recruit participants directly. Data were collected throughout the admission-to-treatment process and later merged to include only cases that resulted in CS. As a quasi-experimental study with an interrupted time series, data were collected at three timepoints: baseline (February–March 2022, prior to intervention implementation), midline (August–September 2022, immediately following implementation), and endline (February–March 2023, six months post-implementation). A total of 50 observations were conducted at each timepoint to assess adherence to written triage protocols.

Structured observation data entailed that for each observed case, a team member documented the patient’s arrival time, the time of initial assessment by a midwife or nurse, and the clinical components of that assessment. These included data such as patients’ assessed vital signs, gestational age, obstetric history, fetal lie, and reasons for referral. The time at which a healthcare provider determined whether an emergency CS was necessary was also recorded. No personal identifiers were collected; instead, each woman was assigned a numeric ID in chronological order, which was used throughout data collection to maintain confidentiality.

Among the observed cases, those who were triaged for potential emergency CS were followed further. Using the unique patient ID, additional data were collected, including the time of decision for CS, the time the procedure was performed, and the clinical indication. Following delivery, maternal and neonatal outcomes were documented, and data on all CS deliveries were disaggregated for further analysis. Data collection occurred consistently across the three timepoints—baseline, midline, and endline—allowing for comparisons.

### 2.4. Data Cleaning and Analysis

We used unique patient IDs to merge all data. The analytic sample was limited to cases that resulted in a CS, meaning that while at each timepoint we followed 50 women referred from RHF to district hospital, we included only those that ended up with a CS. Before excluding any observations with missing data needed to calculate a valid decision-to-delivery interval (DDI), there were 31 cesarean section (CS) cases at baseline, 29 at midline, and 20 at endline. Observations missing either the decision or incision date or time were subsequently excluded, as these cases could not yield a valid DDI value—resulting in the removal of 3 cases from baseline, 1 from midline, and 4 from endline. The final analytic sample therefore included 28 CS cases at baseline, 28 at midline, and 16 at endline.

We used descriptive analyses to summarize patient characteristics, referral indications, and neonatal outcomes at each timepoint (baseline, midline, and endline). For categorical variables, including facility, parity, prior CS, number of fetuses, fetal lie, and referral reasons (e.g., fetopelvic disproportion, hypertensive disorders, uterine scar, fetal distress, malpresentation, ruptured membranes, multigestation, and maternal age younger than 16 or older than 35 years), we calculated frequencies and percentages. We compared the differences across timepoints, baseline and midline, and baseline and endline, using chi-squared tests of independence. For the continuous variable, DDIs, we calculated medians and interquartile ranges (IQRs), and compared distributions across timepoints using the Wilcoxon rank-sum test due to non-normality.

To assess whether the timeliness of cesarean sections improved across timepoints, we examined changes in the median DDIs and the proportion of procedures completed within ≤30 and ≤75 min. Median DDIs were analyzed using quantile regression, adjusting for facility, parity, prior cesarean section, number of fetuses, fetal lie, and all documented reasons for referral. Other potential confounders were initially considered but excluded from the final models due to quasi-complete separation or lack of variability (e.g., no positive cases). The proportions of cases completed within ≤30 and ≤75 min were analyzed using Firth logistic regression models, adjusted for facility, parity, fetal lie, fetopelvic disproportion, and maternal age under 16 or over 35. Firth’s penalized likelihood approach reduces small-sample bias and provides stable estimates in the presence of separation [30,31].

Next, we conducted regression analyses to examine the association between DDI and neonatal death. We used logistic regression to estimate both unadjusted and adjusted ORs for neonatal death, analyzing the interval both as a continuous variable and as binary thresholds (≤30 min and ≤75 min). Multivariable models adjusted for facility, parity, fetal lie, and fetopelvic disproportion. All analyses were conducted using Stata version (18.0, StataCorp LLC, College Station, TX, USA).

### 2.5. Ethics

The study was reviewed and approved by Institutional Review Boards at the University of Michigan (19 March 2021; HUM00195449) and the University of Liberia (5 February 2021; IRB00013730). The study used medical records with the data fully anonymized before analysis. The ethics committee waived the requirement for the informed consent.

## 3. Results

Table 1 presents descriptive data on women who underwent CS at baseline (n = 28), midline (n = 28), and endline (n = 16). Across all timepoints, the majority of women were multiparous, accounting for 64% at baseline, 75% at midline, and 56% at endline. A substantial proportion of those referred for CS also had a history of prior CS: 17.8% at both baseline and midline, and 37.5% at endline. Most women carried singleton pregnancies, although between 10% and 25% presented with breech or transverse fetal lie.

The most frequently documented reasons for referral included fetopelvic disproportion (50% at baseline, 57% at midline, and 25% at endline), prior uterine scar (18%, 21%, and 38%, respectively), ruptured membranes (11% at baseline, 25% at midline, and 0% at endline), multiple gestation (14%, 11%, and 6%, respectively), and maternal age at either extreme—under 16 or over 35 years (21% at baseline, 36% at midline, and 44% at endline).

Median DDI time decreased progressively from baseline to endline, with the median dropping from 170 min (IQR: 101.5–254) at baseline to 111.5 min (61.5–180) at midline, and 53.5 min (22–122) at endline. While the reduction from baseline to midline was not statistically significant (*p* = 0.119), the reduction from baseline to endline was significant (*p* = 0.007). The proportion of cases with incision within 30 min increased from 14.3% at baseline to 10.7% at midline and 37.5% at endline, with marginal significance between baseline and endline (*p* = 0.077). Similarly, the proportion of cases completed within 75 min rose from 21.4% at baseline to 56.3% at endline, with a statistically significant difference (*p* = 0.019).

No maternal deaths were reported across the three timepoints; however, four neonatal deaths (14.29%) were recorded at baseline, three (10.71%) deaths at midline, and one (6.25%) at endline.

Table 2 presents the comparison of DDI at baseline, midline, and endline. Compared to the baseline, the median decision-to-incision interval (DDI) was shorter at both midline and endline, with a non-significant reduction of 42 min at midline (β = −42.0; 95% CI: −142.1, 58.1) and a statistically significant reduction of 117.5 min at endline (β = −117.5; 95% CI: −205.1, −29.9). The odds of having a DDI of ≤30 min did not differ significantly between baseline and either midline (AOR = 0.743; 95% CI: 0.138, 3.979) or endline (AOR = 5.636; 95% CI: 0.527,60.226). However, the odds of achieving a DDI of ≤75 min were significantly higher at endline compared to baseline (AOR = 11.749; 95% CI: 1.320, 104.535), while no significant difference was observed at midline (AOR = 2.408; 95% CI: 0.611, 9.494). These findings suggest substantial improvement in timeliness of CS by endline. These models were adjusted for relevant clinical and facility-level covariates as described in the table footnote.

Table 3 shows the unadjusted and adjusted odds of neonatal deaths based on DDI time thresholds. Neonatal death was not significantly associated with any measure of DDI time. In unadjusted models, both the continuous variable (median minutes) and categorical thresholds (≤30 min and ≤75 min) showed negative but non-significant associations with neonatal death. Specifically, the unadjusted ORs for ≤30 min and ≤75 min were 0.649 (95% CI: 0.072, 5.828) and 0.590 (95% CI: 0.109, 3.1815), respectively. After adjusting for timepoint, facility, parity, fetal lie, and fetopelvic disproportion, the associations remained non-significant, with adjusted ORs of 0.610 (95% CI: 0.028, 13.086) for ≤30 min and 1.185 (95% CI: 0.157, 8.918) for ≤75 min. The adjusted model did not include other variables due to their being dropped from the analysis because of quasi-complete separation or no variation.

## 4. Discussions

Our study demonstrated that implementing a combined MORES and a midwife-led triage program was associated with a substantial and statistically significant reduction in DDI time for emergency CS by endline. Median DDI fell from 170 min at baseline to 53.5 min at endline, and the proportion of cases completed within 75 min more than doubled over the same period. Improvements from baseline to midline trended in the expected direction but were not statistically significant; the most pronounced gains were realized after full implementation and uptake of both interventions. Although neonatal deaths were uncommon and models were underpowered to detect modest effects, adjusted analyses—including Firth logistic regression to address sparse data and quasi-separation for the ≤30 min threshold—did not identify a statistically significant association between shorter DDI and neonatal death. Nonetheless, the direction of effect was protective and clinically meaningful. These findings highlight the potential of integrated referral and triage interventions to address delays in emergency CS, which are well-documented contributors to adverse perinatal outcomes across SSA [20,32]. Future studies should examine the relationship with a larger sample size.

International guidance underscores the urgency of expediting emergency CS, particularly for the most acute indications. Longer DDI windows are generally considered suboptimal when maternal or fetal compromise is suspected [10]. In many LMICs, however, these targets are rarely achieved. Reported median DDIs commonly exceed 2–3 h: Abdulbaki and colleagues in Nigeria documented a median of ~200 min and found 94.5% of emergency cesareans exceeded recommended thresholds, with marked increases in perinatal morbidity and mortality at longer intervals [11]. Similarly, Ayeni et al. reported a mean DDI of ~234 min; nearly all perinatal deaths occurred beyond 90 min, and neonatal morbidity rose with increasing delay [12]. A recent meta-analysis across SSA estimated that only about 6% of CS met the 30 min benchmark and that the mean DDI approached 3 h, further highlighting the system-wide challenge [33]. Against this backdrop, the drop we observed—to a median near one hour and more than half of cases within 75 min at endline—represents a meaningful operational gain.

Emergency obstetric timeliness in resource-constrained settings is shaped by multiple delay points: recognition of severity at the referring facility, communication and transport coordination, clinical prioritization on arrival, and operating-theater readiness [34]. Evidence from a systematic review of referral-strengthening interventions in SSA shows that training, structured triage checklists, and mobile phone–enabled communication can improve recognition of danger signs, referral decisions, inter-facility communication, and perceived maternal outcomes [20]. Our integrated approach combined (a) structured data capture and escalation pathways through MORES—improving situational awareness across facilities and supporting earlier referral—and (b) a midwife-led triage process at the receiving hospital—standardizing intake assessment, case prioritization, and theatre queue management. Together, these strategies likely compressed upstream and on-arrival delays that contribute heavily to prolonged DDI in LMIC settings. Findings from Ethiopia that shorter transfer times, daytime care, and organized clinical teams are strongly associated with meeting recommended DDIs further support the plausibility of our results [35].

Prolonged DDIs in SSA have been linked to structural barriers: workforce shortages, stock-outs, financial hardship, limited transport, and referral bottlenecks [14,15,36]. While it is beyond the scope of this study to examine these factors in detail, it is plausible that such systemic challenges contributed to the findings observed here and are worth investigating more holistically in future studies. CS access itself remains below the 10–15% population coverage associated with mortality reduction in many countries, particularly in rural and lower-resource regions. Multi-country Demographic Health Surveys analyses show wide rural–urban, wealth, and education gradients in cesarean utilization; leveling maternal and child characteristics could substantially narrow these gaps [8,15,37,38,39,40]. Our program was implemented in a setting characterized by referral dependence and variable facility readiness—patterns echoed in Liberia, where fewer than one in five childbirth facilities meet BEmONC standards and referral capacity is limited [15]. By strengthening early identification and triage across linked facilities, MORES and midwife-led triage programs may help mitigate some equity-driven access delays, though financial and geographic barriers remain beyond the scope of the intervention.

Although delayed DDI has been associated with increased perinatal mortality, neonatal sepsis, and the need for higher-level newborn care in several SSA studies, including Nigeria and Uganda reports cited in Abdulbaki, and the Nigeria cohort analyzed by Ayeni et al., our study did not detect a statistically significant relationship between shorter DDI and neonatal death [11,12,41]. Two factors may explain this: first, limited sample size and low event counts reduced statistical power, leading to wide confidence intervals; second, case mix may differ from settings in which fetal distress or abrupt emergencies dominate. We observed directionally protective point estimates for shorter intervals, but models adjusting for facility, parity, fetal lie, and fetopelvic disproportion—and re-estimated using Firth penalized logistic regression to address quasi-separation in the ≤30 min category—remained non-significant. These findings do not rule out clinically important neonatal benefits; rather, they underscore the need for larger, multi-site evaluations powered for perinatal endpoints.

Even absent definitive neonatal mortality effects, reducing DDI is a widely endorsed quality target because it reflects the responsiveness of emergency obstetric systems and may avert progression to fetal asphyxia, infection, and maternal complications [12,20]. Implementation lessons from our combined approach align with broader evidence that team training, structured triage, reliable referral communication, and rapid transfer pathways are feasible leverage points in low-resource settings [18,20,27,35]. We further explored barriers and facilitators related to the adaptation, sustainability, and scale-up of the MORES intervention [42]. Providers reported high perceived usability, fidelity, effectiveness, and scalability of MORES, but noted that technological challenges and the need for consistent staff training must be addressed to optimize implementation [42]. In clinical practice, the findings suggest that investments should prioritize strengthening facility-level response capacity—particularly through routine simulation training, dedicated triage teams, and functional referral and transport systems. Evaluating the effect of these improvements will require integrating continuous data feedback loops (e.g., MORES dashboards), monitoring provider adherence to escalation protocols, and assessing changes in maternal and neonatal outcomes over time. Given persistent inequities in CS access tied to socioeconomic status, geography, and facility capacity across sub-Saharan Africa, linking such quality improvement interventions to broader health financing and system-strengthening strategies will be critical for sustainable impact [8,15,38,39].

### Strengths and Limitations

Strengths of this study include longitudinal measurement across three implementation phases, linkage of referral data with operative timestamps, and use of multiple metrics (continuous DDI, ≤30 min, ≤75 min). Analytically, we applied nonparametric tests appropriate for skewed time distributions and used Firth logistic regression when sparse outcomes produced quasi-separation, improving estimate stability. Limitations include small sample size—particularly at endline—missing or rare data for several referral indication variables (necessitating their exclusion from adjusted models), and limited power. Therefore, the study findings should be interpreted with caution. Additionally, generalizability may be limited due to various factors such as hospital infrastructure, network connectivity, road conditions, staffing, and more. Furthermore, we did not collect additional patient demographic data such as family income, maternal and paternal education level, residence, as well as the facility-based data such as transport interval data or theater staffing metrics.

## 5. Conclusions

In a resource-constrained referral setting, combining a data-driven MORES platform with a midwife-led triage program was associated with marked reductions in DDI time for emergency CS by endline. Although our study was underpowered to demonstrate a corresponding reduction in neonatal mortality, the observed improvements in timeliness advance a critical quality goal linked in other studies to better perinatal outcomes. Potential avenues for future research include investigating long-term maternal and neonatal outcomes and scaling and adapting such integrated referral-triage models to larger, multicenter samples to strengthen emergency obstetric care and narrow equity gaps in CS access across SSA.

## Figures and Tables

**Table 1:** Descriptive statistics on the cesarean sections at baseline, midline, and endline.

	Baseline	Midline	Endline	*p* Value between Baseline and Midline	*p* Value between Baseline and Endline
**Total**	N = 28	N = 28	N = 16		
Facility				0.584	0.392
CB Dunbar	12 (42.86)	10 (35.71)	9 (56.25)		
Phebe	16 (57.14)	18 (64.29)	7 (43.75)		
Parity				0.480	0.750
Multipara	18 (64.29)	21 (75.00)	9 (56.25)		
Nullipara	8 (28.57)	6 (21.43)	5 (31.25)		
Missing	2 (7.14)	1 (3.57)	2 (12.50)		
Previous C/S				0.942	0.128
No	20 (71.43)	19 (67.86)	8 (50.00)		
Yes	5 (17.86)	5 (17.86)	6 (37.50)		
Missing	3 (10.71)	4 (14.29)	3 (14.29)		
Number of fetus				0.267	0.086
Multiples	5 (17.86)	2 (7.14)	-		
Singleton	22 (78.57)	23 (82.14)	14 (87.50)		
Missing	1 (3.57)	3 (10.71)	2 (12.50)		
Fetal Lie				0.155	.601
Breech/Transverse	7 (25.00)	3 (10.71)	3 (18.75)		
Cephalic	17 (60.71)	21 (75.00)	11 (68.75)		
Missing	4 (14.29)	4 (14.29)	2 (12.50)		
**Reasons for referral ^a^**					
Fetopelvic disproportion				0.931	0.038 *
No	10 (35.71)	12 (42.86)	12 (75.00)		
Yes	14 (50.00)	16 (57.14)	4 (25.00)		
Missing	4 (14.29)	-	-		
Hypertension				0.463	0.236
No	22 (78.57)	27 (96.43)	16 (100.00)		
Yes	2 (7.14)	1 (3.57)	-		
Missing	4 (14.29)	-	-		
Prior uterine scar				0.958	0.247
No	19 (67.86)	22 (78.57)	10 (62.50)		
Yes	5 (17.86)	6 (21.43)	6 (37.50)		
Missing	4 (14.29)	-	-		
Fetal distress				0.217	0.408
No	23 (82.14)	24 (85.71)	16 (100.00)		
Yes	1 (3.57)	4 (14.29)	-		
Missing	4 (14.29)	-	-		
Fetal malpresentation				0.219	0.501
No	18 (64.29)	24 (85.71)	13 (81.25)		
Yes	7 (25.00)	4 (14.29)	3 (18.75)		
Missing	3 (10.71)	-	-		
Rupture of membranes				0.254	0.141
No	21 (75.00)	21 (75.00)	16 (100.00)		
Yes	3 (10.71)	7 (25.00)	-		
Missing	4 (14.29)	-			
Multi gestation				0.531	0.329
No	20 (71.43)	25 (89.29)	15 (93.75)		
Yes	4 (14.29)	3 (10.71)	1 (6.25)		
Missing	4 (14.29)	-	-		
Age < 16 or >35				0.404	0.215
No	18 (64.29)	18 (64.29)	9 (56.25)		
Yes	6 (21.43)	10 (35.71)	7 (43.75)		
Missing	4 (14.29)	-	-		
**Decision- to- incision** **interval**					
Median (25%, 75%)	170 (101.5, 254)	111.5 (61.5, 180)	53.5 (22, 122)	0.119	0.007 **
≤30 min, n (%)	4 (14.29)	3 (10.71)	6 (37.5)	0.686	0.077
≤75 min, n (%)	6 (21.43)	10 (35.71)	9 (56.25)	0.237	0.019 *
**Outcomes**					
Maternal outcome				/	/
Dead	-	-	-		
Alive	27 (96.43)	27 (96.43)	14 (87.50)		
Missing	1 (3.57)	1 (3.57)	2 (12.50)		
Neonatal outcome				0.685	0.435
Dead	4 (14.29)	3 (10.71)	1 (6.25)		
Alive	23 (82.14)	24 (85.71)	14 (87.50)		
Missing	1 (3.57)	1 (3.57)	2 (12.50)		

^a^ Anemia, infections, hemorrhage, and uterine rupture were excluded from the reasons for referral due to the absence of any positive cases. All percentages were calculated with missing observations in consideration. All variables were categorical and compared across timepoints using the chi-squared test, except for the median minimum decision-to-incision time, which was a skewed continuous variable and analyzed using the Wilcoxon rank-sum test. * *p* value < 0.05; ** *p*-value significant at <0.05.

**Table 2:** Comparison of Decision-to-Incision Interval Outcomes Between Baseline and Midline, and Baseline and Endline.

	Baseline and Midline	Baseline and Endline
**Decision-to-incision interval**		
Median, β (95% CI)	−42.0 (–142.118, 58.118)	−117.5 (−205.050, −29.949) *
≤30 min, AOR (95%CI)	0.743 (0.138, 3.979)	5.636 (0.527,60.226)
≤75 min, AOR (95%CI)	2.408 (0.611, 9.494)	11.749 (1.320, 104.535) *

β: Coefficient; AOR: Adjusted Odds Ratio; CI: Confidence Interval. * *p*-value significant at <0.01. Quantile regression models for median decision-to-incision time were adjusted for facility, parity, prior cesarean section, number of fetuses, fetal lie, and all reasons for referral. Firth logistic regression models for ≤30 and ≤75 min outcomes were adjusted for facility, parity, fetal lie, fetopelvic disproportion, and maternal age under 16 or over 35.

**Table 3:** Unadjusted and adjusted odds of neonatal deaths based on decision to incision time thresholds.

	Neonatal Death	
**Decision-to-incision interval**	Odds Ratio (95% CI)	Adjusted Odds Ratio (95% CI)
Median (model 1)	1.001 (0.997, 1.004)	1.001 (0.997, 1.005)
≤30 min (model 2)	0.649 (0.072, 5.828)	0.610 (0.028, 13.086)
≤75 min (model 3)	0.590 (0.109, 3.1815)	1.185 (0.157, 8.918)

AOR: Adjusted Odds Ratio; CI: Confidence Interval. Adjusted odds ratios are adjusted for timepoints, facility, parity, fetal lie, fetopelvic disproportion, and maternal age younger than 16 or older than 35. Three separate models were ran for the adjusted model with three different independent min decision-incision variables.

## Data Availability

The datasets generated during or analyzed during this study are available from the corresponding author on reasonable request.
